# Validation of Potential Reference Genes for qPCR in Maize across Abiotic Stresses, Hormone Treatments, and Tissue Types

**DOI:** 10.1371/journal.pone.0095445

**Published:** 2014-05-08

**Authors:** Yueai Lin, Chenlu Zhang, Hai Lan, Shibin Gao, Hailan Liu, Jian Liu, Moju Cao, Guangtang Pan, Tingzhao Rong, Suzhi Zhang

**Affiliations:** Key Laboratory of Biology and Genetic Breeding of Maize in Southwest China of Agricultural Department, Maize Research Institute, Sichuan Agricultural University, Chengdu, China; Rush University Medical Center, United States of America

## Abstract

The reverse transcription quantitative polymerase chain reaction (RT-qPCR) is a powerful and widely used technique for the measurement of gene expression. Reference genes, which serve as endogenous controls ensure that the results are accurate and reproducible, are vital for data normalization. To bolster the literature on reference gene selection in maize, ten candidate reference genes, including eight traditionally used internal control genes and two potential candidate genes from our microarray datasets, were evaluated for expression level in maize across abiotic stresses (cold, heat, salinity, and PEG), phytohormone treatments (abscisic acid, salicylic acid, jasmonic acid, ethylene, and gibberellins), and different tissue types. Three analytical software packages, geNorm, NormFinder, and Bestkeeper, were used to assess the stability of reference gene expression. The results revealed that elongation factor 1 alpha (*EF1α*), tubulin beta (*β-TUB*), cyclophilin (*CYP*), and eukaryotic initiation factor 4A (*EIF4A*) were the most reliable reference genes for overall gene expression normalization in maize, while *GRP* (Glycine-rich RNA-binding protein), *GLU1*(beta-glucosidase), and *UBQ9* (ubiquitin 9) were the least stable and most unsuitable genes. In addition, the suitability of *EF1α*, *β-TUB*, and their combination as reference genes was confirmed by validating the expression of *WRKY*50 in various samples. The current study indicates the appropriate reference genes for the urgent requirement of gene expression normalization in maize across certain abiotic stresses, hormones, and tissue types.

## Introduction

A determination of the gene expression pattern is very important for the functional exploration of a target gene. The reverse transcription quantitative polymerase chain reaction (RT-qPCR) is a preferred technique for the detection and quantification of gene expression due to its sensitivity, specificity, dynamic range, and high throughput capacity [1]–[3]. However, to avoid the bias caused by the RNA samples, reverse transcription, and polymerase chain reaction, as well as to assure the accuracy and reliability of gene expression analysis during the RT-qPCR process, it is essential to normalize the data using appropriate reference genes prior to evaluating the expression patterns in biological samples [4]–[6]. The expression of reference genes is assumed to be stable and constitutive across all experimental designs regardless of treatment, tissue, or species [7], [8]. However, the commonly used reference genes, such as glyceraldehyde-3-phosphate dehydrogenase (*GAPDH*), elongation factor 1*α* (*EF1α*), tubulin *β*-chain (*β-TUB*), polyubiquitin (*UBQ*), 18S ribosomal RNA (18S rRNA), and *β*-actin (*ACT*), should be carefully handled as internal controls, because their transcript levels are variable under particular experimental conditions [2], [3], [7], [9]–[12]. These existing reference genes are limited to particular experimental conditions and designs. The use of reference genes whose normalization has not been validated will lead to erroneous gene expression profiles for the target gene. Therefore, it is necessary to evaluate potential reference genes under different experimental conditions prior to their use in RT-qPCR data normalization.

Statistical algorithms such as geNorm [13], NormFinder [14], and BestKeeper [15] are well developed and are widely used to assess the expression stability of reference genes. Among these algorithms, geNorm provides not only stability rankings but also the effective number of reference genes. In recent years, an increasing number of studies on reference gene selection have been performed in plant species including rice [16], [17], soybean [18]–[20], wheat [21], [22], cotton [23], [24], sugarcane [25], citrus [26],tomato [27], [28], coffee [29], [30], potato [31], banana [32], peach [33], grapevine [34], sunflower [35], tobacco [36], and radish [37].

Maize, one of the most important cereal crops, also plays a growing role in industry and energy resources. However, major global abiotic stresses such as drought, heat, cold, and salinity cause huge reductions in the production of corn every year. Additionally, hormones have been implicated in the plant response to numerous abiotic and biotic stresses, as well as developmental processes [38]–[42]. An increasing number of researchers are concentrating on the complicated regulatory network of stresses and hormone signaling. Nevertheless, despite the rapid exploration of the maize genome and the growing requirement for the deep biological understanding of gene function aided by gene expression patterns, very limited information is available on the expression stability of reference genes in maize under particular experimental conditions. Manoli *et al*. reported the identification of five novel reference genes in maize from microarray data gathered under a series of conditions including +N/−N nutrient, day/night cycle, darkness, and high temperature [43]. However, the traditionally used reference genes are still widely employed and are the preferred choices for gene expression normalization. Moreover, a general lack of information remains regarding the suitability of reference genes in maize across hormones and abiotic stresses, to which researchers already pay more attention. Therefore, the selection of suitable reference genes under these experimental conditions is an urgent requirement. In the present study, we evaluated the expression stability of ten potential reference genes, eight of which (*GAPDH*, *EF1α*, *ACT2*, *β-TUB*, *UBQ9*, *CYP*, *EIF4A*, and *UBQ7*) were commonly used internal control genes and two of which (*GLU1* and *GRP*) were novel candidate reference genes identified from our microarray datasets of salt and ABA treatments, respectively, in a set of 26 maize samples collected from different experimental conditions with respect to abiotic stresses (salt, heat shock, cold, and PEG), hormones and tissue types. Furthermore, the transcription factor *WRKY50* was investigated to test the usefulness of the selected reference genes in expression analysis. The results indicated several validated reference genes suitable for RT-qPCR analysis in maize under certain experimental conditions and clearly demonstrated that different reference genes should be validated according to the particular experimental conditions.

## Materials and Methods

### Plant sample preparation

The maize inbred line 178 was used for all the experimental treatments. The seeds were sterilized with 0.1% NaClO for 30 min and then washed three times with sterile water. The seeds were germinated on filter paper saturated with water in complete darkness at 28±1°C. After 3 days, seedlings were grown in a 1/4 strength aerated Hoagland solution in the greenhouse under a 16/8-h (light/dark) photocycle at 28/26°C (day/night). Seedlings at the three leaf stage were used for the abiotic stress and hormone treatments or for the harvesting of different plant tissues (root, stem and leaf).

### Abiotic stress and hormone treatments

For the cold and heat treatments, seedlings at the three leaf stage were incubated at 4°C or 42°C for 0, 2, 6, 12, and 24 h. For the drought treatment, the seedlings were treated with 20% PEG6000 and collected at various time intervals (0, 12, 24, 48, and 72 h). For the salinity treatment, the seedlings were subjected to 200 mM NaCl and harvested at 0, 12, 24, 48, and 72 h. For the hormone treatments, the seedlings were subjected to 100 µM hormone solutions of salicylic acid (SA), jasmonic acid (JA), abscisic acid (ABA), gibberellins (GA), and 1-aminocyclopropane-1-carboxylic acid (ACC) for 12 h. All the collected samples were frozen in liquid nitrogen immediately after harvest and stored at −80°C until RNA extraction.

### Total RNA extraction and cDNA synthesis

Total RNA was extracted with Trizol reagent (TaKaRa, Dalian, China) according to the manufacturer's instructions. To avoid genomic DNA contamination, 1 µg sample RNA was treated with 2 U DNase I (Takara) for 30 min at 37°C before reverse transcription (RT); the DNase I was inactivated by incubating the samples at 85°C for 10 min. The concentration of each RNA sample was measured using NanoVue Plus; only the RNA samples with a 260/280 ratio between 1.9 and 2.1 and a 260/230 ratio (an indication of reagent contamination) greater than 2.0 were used for further analysis. The integrity of the RNA samples was also assessed by 1% (w/v) agarose gel electrophoresis with ethidium bromide staining. First-strand cDNA synthesis was conducted using the Prime-Script RT reagent Kit (TaKaRa, Dalian, China) according to the manufacturer's instructions.

### Quantitative RT-PCR

RT-qPCR was conducted in 96-well plates and performed on the Bio-Rad CFX96 real-time PCR System (Bio-Rad, CA) under universal cycling conditions (95°C for 1 min, 40 cycles of 95°C for 5 s, and 60°C for 30 s). Each reaction mix contained 1 µl diluted cDNA, 5 µl 2×Power SYBR Premix Ex Taq II (Takara), 3 µl RNase free water, and 0.5 µM of each primer, for a final volume of 10 µl. A no-template control was also included in each run for each gene. Each sample was conducted in technical triplicates with at least two biological replicates. In addition, melting curves were generated at 65–95°C after 40 cycles to check for primer specificity.

### Statistical Analyses

To select a suitable reference gene, the stability of the mRNA expression of each reference gene was statistically analyzed with three software packages: geNorm [13], NormFinder [14], and BestKeeper [15]. All three software packages were used according to the manufacturer's procedures. For geNorm, the raw Ct values were transformed into the required data input format. The maximum expression level of each gene was used as a control and was set to a value of 1. Relative expression levels were then calculated from the Ct values using the following formula: 2^−ΔCt^ (ΔCt = each corresponding Ct value−minimum Ct value). The geNorm algorithm further calculated the expression stability value (M) for each gene and the pairwise variation (v) of that gene with the others. All of the tested genes were ranked according to their M values, and the number of reference genes necessary for optimal normalization was also indicated. NormFinder used the same input file format as geNorm, while the BestKeeper analyses were based on the untransformed Ct values.

### Normalization of *WRKY50*

The maize transcription factor *WRKY50* was used as the target gene. The expression levels of *WRKY50* were quantified across the samples treated by abiotic stresses for 24 h or hormones for 12 h and the different tissue types using both of the individual stable reference genes determined by geNorm and their combination. Information on the primers for *WRKY50*, such as melting curve, standard curve, and specificity, are shown in Figure A and B in Figure S4 and Table S2.

## Results

### Amplification specification and PCR efficiency

In order to investigate the specificity of the primers for the reference genes designed in the current study, agarose gel electrophoresis and melting curve analyses were performed in maize seedlings. The specificity of the primers was supported by the presence of a single band of the expected size after amplification on the agarose gel (Figure S1) and further confirmed by the presence of a single peak during the melting curve analysis and sequencing analysis (Figure A–J in Figure S2 and Text S1) before performing the RT-qPCR experiment. The amplification efficiency of each primer pair was estimated using the LinRegPCR 12.5 program and is shown in Table 1. A standard curve was first established using a 10-fold serial dilution of cDNA before the calculation of the gene-specific PCR efficiency. The gene-specific PCR efficiency (E) and the regression coefficient (R^2^) were calculated using the slope of the standard curve (Figure A–J in Figure S3). The linear R^2^ for the primers ranged between 0.998 and 1.000 over 1000 fold of cDNA dilution. Additionally, the PCR efficiencies of the primers ranged from 93.1% to 102.6% (Table 1).

**Table 1:** Descriptions of candidate reference genes, their primer sequences, product sizes and amplicon characteristics.

Gene symbol	Accession number	Primer sequence (5′–3′)	Tm(°C)	Size (bp)	PCR efficiency	R^2^
*GAPDH*	X07156	F:CCATCACTGCCACACAGAAAAC	62.82	170	102.6%	0.999
		R:AGGAACACGGAAGGACATACCAG	63.64			
*EF1α*	NM_001112117	F: TGGGCCTACTGGTCTTACTACTGA	61.37	135	95.7%	1.000
		R: ACATACCCACGCTTCAGATCCT	62.13			
*β-TUB*	NP_001105457	F: CTACCTCACGGCATCTGCTATGT	62.74	139	97.4%	0.998
		R: GTCACACACACTCGACTTCACG	61.84			
*ACT2*	NM_001154731	F: CTGAGGTTCTATTCCAGCCATCC	63.31	133	93.1%	0.998
		R: CCACCACTGAGGACAACATTACC	62.80			
*UBQ9*	NM_001138130	F: TACAGTTCTACAAGGTGGACGAC	63.73	119	94.0%	0.998
		R: GCAGTAGTGGCGGTCGAAGT	62.71			
*CYP*	M55021	F: CTGAGTGGTGGTCTTAGT	59.3	100	98.7%	1.000
		R: AACACGAATCAAGCAGAG	59.1			
*EIF4A*	NM_001111902	F: CGTCCAGAGGTTCTACAA	59.7	183	98.6%	0.999
		R: CATCCTTCGCCACAATAC	59.7			
*UBQ7*	NM_001153555	F: CAGACTACAACATCCAGAAG	59.3	156	99.0%	0.999
		R: TATTAGACGACGACATCCATA	59.7			
*GLU1*	NM_001111984	F: ATGAAGGAGTCTGCCAAGTG	63.9	196	97.2%	0.999
		R: CGGTGCTGGAGAGTATGC	64.1			
*GRP*	X12564	F: AACGAGTCGCTGGAGAAT	62.5	116	94.2%	0.999
		R: TCGGAGGAGAAGGTAACG	61.9			

### Expression Profiles of Reference Genes

**Figure 1:**
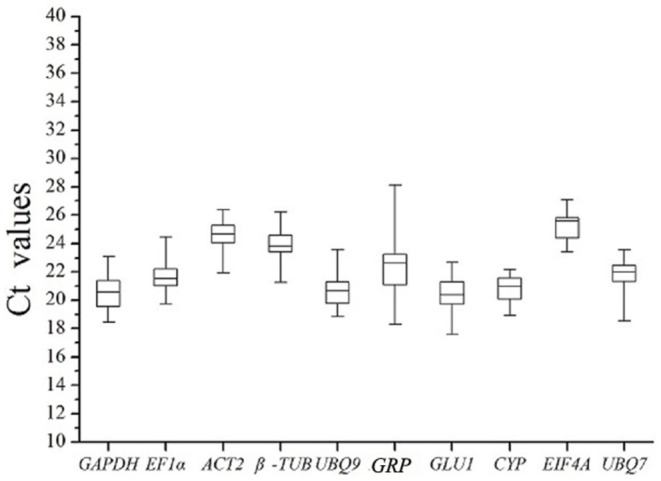
Range of Ct values of the candidate reference genes obtained from all samples. Each box corresponding to *GAPDH*, *EF1α*, *ACT2*, *β-TUB*, *UBQ9*, *GRP*, *GLU1*, *CYP*, *EIF4A* and *UBQ7* indicates the 25% and 75% percentiles. Whiskers represent the maximum and minimum values. The median is depicted by the line across the box.

The cycle threshold (Ct) value reflects the cycle number at which the fluorescence generated within a reaction crosses the threshold. Expression profile analysis allowed for a straight and visual assessment of those reference genes that had a narrow Ct range in all samples across all experimental conditions. All samples across all experimental conditions were used to analyze the expression profiles of the candidate reference genes by RT-qPCR. During the melting curve analysis, a unique dissociation curve was observed for all amplicons (Figure A–J in Figure S2) after 40 cycles of RT-qPCR. The Ct values for the ten reference genes ranged from 18 to 28, and the majority of the Ct values were between 20 and 25 (Figure 1). The least variable Ct value indicted the most stable gene. *GRP* exhibited large variances in its expression levels, and its Ct values differed by over 8.0 across the heat-treated sample set. Conversely, *GAPDH*, *EF1α*, *ACT2*, *EIF4A*, and *CYP* all showed a narrow range of mean Ct values in their respective expression levels, indicating that these genes were more stably expressed than the others. However, the simple comparison of raw Ct values is insufficient for the evaluation of the expression stability of candidate reference genes. To obtain accurate gene expression data, this approach must be combined with other methods to select a set of reliable reference genes for the normalization of gene expression under certain conditions.

### GeNorm Analysis

**Figure 2:**
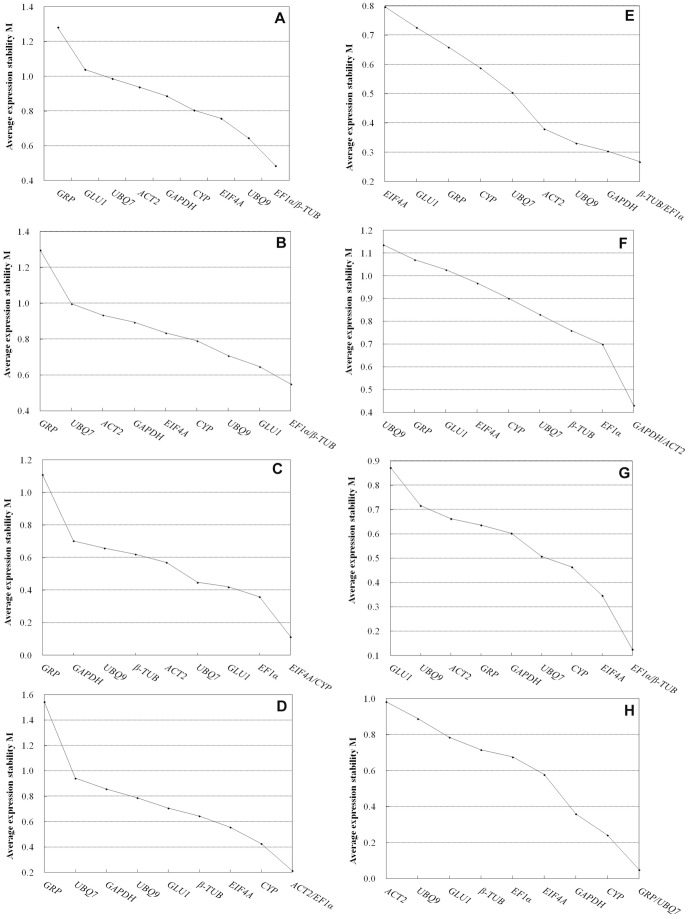
Gene expression stability and ranking of potential reference genes as calculated by geNorm. Samples are treated or directly harvested from the experimental conditions: (A) total, (B) abiotic stresses, (C) cold, (D) heat, (E)PEG, (F)salinity, (G) hormones, (H) different tissues.

GeNorm software [13] was used to analyze the expression stability of the tested genes in the various samples and to rank them accordingly. A lower M value indicates that the reference gene is more stably expressed and is more suitable as a control gene. As shown in Figure 2, we analyzed the expression profile data obtained from eight experimental sets in maize. Each gene in this study had a relatively low M value, less than the default cutoff value of 1.5 suggested by geNorm (Table S1). *CYP* and *EIF4A* were the most stably expressed genes under cold stress, with an M value of 0.114 (Figure 2C). For the heat-treated samples, *EF1α* and *ACT2* were ranked as the most stable (M = 0.214) (Figure 2D). For the PEG-treated samples, *β-TUB* and *EF1α* were the most stable, with an M value of 0.268 (Figure 2E); these genes were also most stable for the hormone-treated samples, with M values of 0.125 (Figures 2G). For the NaCl-treated samples, *GAPDH* and *ACT2* perfected best (M = 0.431) (Figure 2F). For the tissue-specific samples, *GRP* and *UBQ7* expressed most stably, with an M value of 0.05 (Figure 2H). However, *GRP*, *GLU1*, and *UBQ9* displayed high M values under most experimental conditions, suggesting that these genes expressed less stably. Additionally, in the context of the total sample set or the sample set of the abiotic stresses (composed of PEG, heat, and cold), *EF1a* and *β-TUB* ranked as the most stable, with M values of 0.485 and 0.549, respectively (Figure 2A, 2B). Therefore, these two reference genes were deemed the most suitable for the widest range of test conditions in the current study.

**Figure 3:**
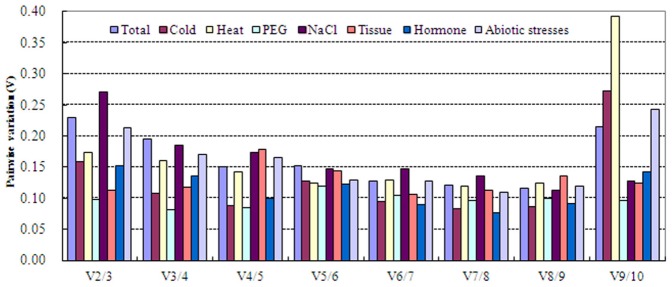
Determination of the optimal number of reference genes for normalization by pairwise variation using geNorm.

The geNorm software determines the optimal number of genes for accurate normalization by calculating the pairwise variation (V) using a normalization factor (NF). A lower pairwise variation signifies a better combination of genes for reference. A variation of <0.15 indicates that an additional reference gene makes no significant contribution to the normalization factor. For the PEG-treated samples and the different tissues, the V_2/3_ values of 0.098 and 0.112, respectively, indicated that the inclusion of a third reference gene did not contribute significantly to the variation of the normalization factor, as these values were less than the cutoff value of 0.15 (Figure 3 and Table S1). This result reveals that the two most stable reference genes, *GRP* and *UBQ7* for the different tissues and *EF1α* and *β-TUB* for the PEG-treated samples, are sufficient for reliable normalization under these conditions. However, under the cold and hormone treatments, the pairwise variation of V_2/3_ was greater than 0.15 (0.159 and 0.152, respectively), while that of V_3/4_ was less than 0.15 (0.107 and 0.136, respectively), indicating that three reference genes (*CYP*, *EIF4A*, and *EF1α* for cold stress; *EF1a*, *β-TUB*, and *EIF4A* for hormone treatment) were necessary to normalize gene expression reliably (Figure 3). According to this principle, geNorm analysis indicated that four reference genes were appropriate for gene expression normalization under heat treatment (*EF1α*, *ACT2*, *CYP*, and *EIF4A*, V_4/5_ = 0.142), five reference genes were appropriate under NaCl treatment (*GAPDH*, *ACT2*, *EF1a*, *β-TUB*, *UBQ7*, V_5/6_ = 0.148) and abiotic stress (*EF1a*, *β-TUB*, *GLU*, *UBQ*9, *CYP*, *EIF4A*, V_5/6_ = 0.129), and six reference genes were appropriate across all experimental conditions (*EF1a*, *β-TUB*, *UBQ9*, *EIF4A*, *CYP*, *GAPDH*, *ACT2*, V_6/7_ = 0.148) (Table S1).

### NormFinder Analysis

Similarly to geNorm, the NormFinder program also determines the expression stabilities of reference genes. The genes with the lowest M values are the most stably expressed. The outputs of the NormFinder analysis were very similar to those of geNorm, as shown in Table 2. NormFinder analysis also identified that *EF1α* and *β-TUB* were the most stably expressed genes under most experimental conditions, but slight differences in the ranking order were indicated in some cases, such as the samples treated by PEG and abiotic stresses (the ranking changing from 1^st^ in geNorm to 3^rd^ in NormFinder) and NaCl (the ranking changing from 3^rd^ and 4^th^ to 1^st^ and 2^nd^, respectively). For the case of the different tissues, although the top two reference genes (*UBQ7* and *GRP*) indicated by the NormFinder method differed from the two identified by the geNorm method, the results calculated by geNorm and BestKeeper were virtually identical hereinafter for the different tissues (Table 3). Overall, the stable reference genes identified by NormFinder were highly consistent with those obtained from geNorm analysis.

**Table 2:** Ranking of candidate reference genes according to their expression stability value calculated by NormFinder.

Rank	Total	Cold	Heat	PEG	NaCl	Tissue	Abiotic stresses	Hormone
1	*EF1α*	*EF1α*	*EF1α*	*β-TUB*	*EF1α*	*GAPDH*	*EF1α*	*β-TUB*
M value	0.169	0.106	0.074	0.103	0.23	0.045	0.19	0.123
2	*β-TUB*	*EIF4A*	*ACT2*	*GAPDH*	*β-TUB*	*EIF4A*	*CYP*	*EF1α*
M value	0.324	0.167	0.074	0.109	0.282	0.203	0.343	0.135
3	*CYP*	*GLU1*	*CYP*	*EF1α*	*GAPDH*	*CYP*	*β-TUB*	*EIF4A*
M value	0.363	0.173	0.28	0.215	0.444	0.304	0.35	0.23
4	*EIF4A*	*UBQ7*	*GLU1*	*ACT2*	*EIF4A*	*EF1α*	*EIF4A*	*UBQ7*
M value	0.445	0.192	0.369	0.231	0.453	0.35	0.499	0.292
5	*ACT2*	*CYP*	*β-TUB*	*UBQ9*	*CYP*	*β-TUB*	*GLU1*	*CYP*
M value	0.524	0.206	0.434	0.31	0.529	0.441	0.505	0.335
6	*UBQ7*	*ACT2*	*EIF4A*	*CYP*	*ACT2*	*GLU1*	*ACT2*	*ACT2*
M value	0.575	0.412	0.439	0.405	0.698	0.503	0.507	0.354
7	*UBQ9*	*β-TUB*	*UBQ7*	*UBQ7*	*GRP*	*UBQ7*	*UBQ7*	*GRP*
M value	0.633	0.414	0.519	0.503	0.764	0.573	0.623	0.492
8	*GAPDH*	*UBQ9*	*UBQ9*	*GRP*	*UBQ7*	*GRP*	*UBQ9*	*GAPDH*
M value	0.673	0.542	0.751	0.546	0.824	0.575	0.651	0.513
9	*GLU1*	*GAPDH*	*GAPDH*	*GLU1*	*UBQ9*	*ACT2*	*GAPDH*	*UBQ9*
M value	0.724	0.706	0.95	0.617	0.861	0.85	0.68	0.56
10	*GRP*	*GRP*	*GRP*	*EIF4A*	*GLU1*	*UBQ9*	*GRP*	*GLU1*
M value	1.478	1.884	2.712	0.651	0.956	0.891	1.668	0.983

**Table 3:** Ranking of candidate reference genes in order of their expression stability as calculated by BestKeeper.

Rank	Cold	Heat	PEG	NaCl	Total	Tissue	Abiotic stresses	Hormone
1	*EIF4A*	*ACT2*	*β-TUB*	*EIF4A*	*EF1α*	*GRP*	*EF1α*	*EF1α*
CV±SD	1.62±0.41	1.60±0.41	0.97±0.23	2.91±0.77	3.00±0.65	1.98±0.47	2.82±0.61	1.51±0.32
2	*CYP*	*EF1α*	*GAPDH*	*CYP*	*ACT2*	*UBQ7*	*CYP*	*β-TUB*
CV±SD	1.91±0.40	1.88±0.42	1.21±0.24	3.87±0.84	3.15±0.77	2.20±0.48	3.01±0.63	1.61±0.38
3	*UBQ9*	*UBQ7*	*ACT2*	*EF1α*	*EIF4A*	*CYP*	*ACT2*	*EIF4A*
CV±SD	2.34±0.47	2.39±0.54	1.26±0.31	3.96±0.89	3.17±0.80	3.52±0.72	3.08±0.76	1.84±0.47
4	*ACT2*	*EIF4A*	*EF1α*	*UBQ9*	*β-TUB*	*GAPDH*	*EIF4A*	*GRP*
CV±SD	2.38±0.60	2.42±0.63	1.58±0.34	4.44±0.95	3.26±0.78	4.17±0.91	3.08±0.78	2.12±0.49
5	*β-TUB*	*β-TUB*	*UBQ9*	*β-TUB*	*CYP*	*ACT2*	*β-TUB*	*ACT2*
CV±SD	2.68±0.63	2.54±0.62	1.92±0.40	4.44±1.10	3.29±0.69	4.19±1.02	3.37±0.81	2.56±0.62
6	*EF1α*	*UBQ9*	*CYP*	*GRP*	*UBQ7*	*EIF4A*	*UBQ9*	*UBQ7*
CV±SD	2.96±0.63	3.15±0.66	2.28±0.47	4.83±1.12	3.97±0.87	5.26±1.32	3.86±0.80	2.67±0.59
7	*GAPDH*	*GLU1*	*UBQ7*	*GAPDH*	*UBQ9*	*β-TUB*	*UBQ7*	*GAPDH*
CV±SD	3.37±0.68	3.30±0.70	2.39±0.52	4.86±1.03	4.29±0.89	5.42±1.32	4.41±0.96	3.01±0.63
8	*UBQ7*	*CYP*	*EIF4A*	*ACT2*	*GAPDH*	*EF1α*	*GLU1*	*CYP*
CV±SD	3.44±0.76	3.57±0.75	2.59±0.64	5.29±1.32	4.87±1.01	6.35±1.42	4.87±1.01	3.09±0.66
9	*GLU1*	*GAPDH*	*GRP*	*GLU1*	*GLU1*	*GLU1*	*GAPDH*	*UBQ9*
CV±SD	3.69±0.74	4.43±0.95	2.91±0.65	6.28±1.34	5.48±1.11	7.98±1.54	5.06±1.04	3.24±0.67
10	*GRP*	*GRP*	*GLU1*	*UBQ7*	*GRP*	*UBQ9*	*GRP*	*GLU1*
CV±SD	8.91±2.05	11.96±2.59	3.56±0.73	7.05±1.58	6.71±1.52	9.06±1.88	7.71±1.72	5.15±1.01

### BestKeeper Analysis

The BestKeeper index is based on the average Ct values of each duplicated reaction. For analysis using BestKeeper, the variation in gene expression is calculated based on the standard deviation (SD) and coefficient of variance (CV) [15]. The most stable genes are identified as those which exhibit the lowest coefficients of variance and standard deviations (CV±SD). Any proposed reference gene with a SD>1 is considered as inconsistent and should be excluded. In this study, the ranking of the candidate reference genes was compatible with the outputs obtained from geNorm and NormFinder. *EF1α* and *β-TUB* remained the two most stable reference genes in the hormone-treated sample, but their ranking slightly changed in the total sample and the abiotic stress and PEG-treated samples. Nevertheless, the lower CV±SD values of *EF1α* and *β-TUB* again identified them as among the most stable candidate reference genes. This result was consistent with those obtained using geNorm and NormFinder. The two most stable genes (*GRP* and *UBQ7*) among the different tissues corresponded with those indicated by geNorm but were different from those calculated by NormFinder. Additionally, geNorm and NormFinder selected similar stable reference genes for the NaCl-treated sample set, while BestKeeper analysis ranked *EIF4A* and *CYP* as the two most stable reference genes. These slight differences may have been caused by the distinct statistical algorithms of the three methods. However, it is notable that *GRP, GLU1*, and *UBQ9* were among the least stably expressed genes identified by BestKeeper, as was consistent with the results obtained using geNorm and NormFinder (Table 3). Due to its higher values of M and CV in all three algorithm methods, *GAPDH* could also be designated as an unstably expressed reference gene except in the PEG- and NaCl-treated samples and the different tissues.

### Reference genes validation

**Figure 4:**
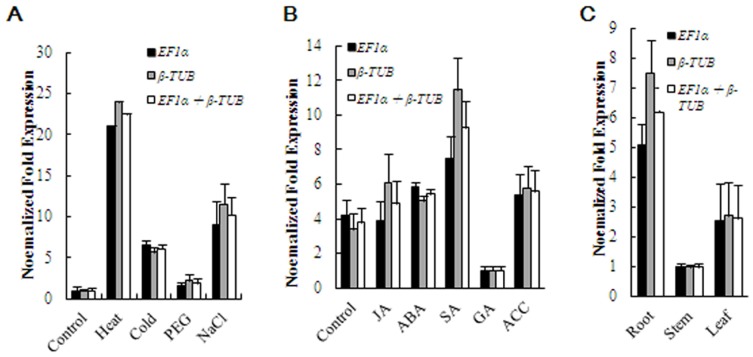
Relative quantification of *WRKY50* expression using the selected reference gene(s). Relate expression of *WRKY50* was normalized using the single most stable reference gene *EF1α*, *β-TUB* and their combination *EF1α* +*β-TUB* in sample sets across (A) abiotic stresses, (B) hormone application, and (C) different tissue types.

To test the effect of reference gene selection on the outcome of a practical experiment, we further validated the relative expression patterns of the transcription factor *WRKY50* in maize, using the most stable reference genes *EF1a* and *β-TUB* and their combination (*EF1a*+*β-TUB*), across samples treated by abiotic stresses for 24 h or hormones for 12 h and taken from different tissue types (Figure 4). In *Arabidopsis*, this gene can be induced by bacteria [44], [45] and chitooctaose [46]. Sekhon *et al.* recently found that maize *WRKY50* is expressed in a tissue-specific manner at different developmental stages, according to microarray analysis [47]. We also found that *WRKY50* was upregulated in maize in response to a number of stresses (Lin *et al.*, unpublished data). The target gene is assumed to have consistent expression patterns irrespective of the reference genes used for normalization, and this was the case observed for *WRKY50*. In the current study, the transcript abundance of *WRKY50* increased significantly in the heat-treated samples, moderately in the cold- and NaCl-treated samples, and weakly in the PEG-treated samples. Meanwhile, the *WRKY50* expression remained unaltered in most hormone-treated samples except for its upregulation in SA-treated samples and downregulation in GA-treated samples. These results were in accordance with the behavior of the WRKY transcription factor, which is involved in stress and hormone responses, previously described in numerous reports [48]–[52]. In terms of the tissue types, the expression patterns of *WRKY50* were consistent with those reported by Sekhon et al. [47], who found the highest transcript abundance in the root, followed by the leaf and stem, in the earlier stages of maize development. The relative expression profiles for *WRKY50* were very similar across the experimental sets when normalized using either *EF1a*, *β-TUB*, or *EF1a*+*β-TUB*, although at slightly different levels (Figure 4). However, as Figure 4 shows, the relative transcript abundance for *WRKY50* was dependent on the reference gene(s) used for normalization. When the expression of *WRKY50* was normalized using a combination of *EF1a* and *β-TUB*, identified by geNorm as most stable reference genes, the fold expression of *WRKY50* was between those obtained using either *EF1a* or *β-TUB* as the reference gene. This result clearly indicated that the use of more than one reference gene for normalization provided a more accurate representation of target gene expression when tested across variable experimental conditions and reinforced the importance of reference gene validation prior to experimental application.

## Discussion

Maize, one of the most important food crops, also plays an important role in industry and energy production. However, despite the rapid exploration of the maize genome and the growing requirement for the deep biological study of gene function, very limited information is available on the expression stability of reference genes in maize under certain experimental conditions. The expression pattern, a reflection of the biological function of a target gene, is preferably detected by RT-qPCR method, in which reference gene is used for normalization. The expression patterns of reference genes are expected to be stable irrespective of experimental conditions. However, the variability of the expression patterns of reference genes reported in previously studies has emphasized that the selection of suitable reference genes is a necessary to avoid bias in gene expression profiles and imprecise or incorrect results. geNorm, NormFinder and BestKeeper are three popular algorithms used to evaluate the stability of reference genes, each according to different parameters. The rankings of gene stability obtained using these algorithms were largely consistent, although the order of the top reference genes did vary between them, as did the order of the most unstable reference genes in many previous reports[11], [22], [33]–[34], [53].

In this study, the expression stability of ten candidate reference genes in maize was systematically assessed by the three algorithms geNorm, NormFinder, and BestKeeper to determine suitable internal control genes for studies on abiotic stresses (cold, heat, NaCl, and PEG), hormones, and different tissue types. The concentration, temperature or developmental stage for sample collection in each treatment were those that most widely used in experimental designation. Meanwhile, the application of various time courses or different tissue types for sample collection under certain treatment condition provides more accurate and appropriate criterion for the selection of suitable reference genes than previous reports which used only one time point or tissue type. According to the M values of the geNorm method, *EF1α* and *β-TUB* were the most stable reference genes in the total samples and the abiotic stressed samples, as well as in the PEG- and hormone-treated samples, which was consistent with the results of NormFinder and BestKeeper. For the cold-treated samples and the different tissue samples, the top two reference genes determined by geNorm were the same as those assigned by BestKeeper, but not those identified by NormFinder. In case of the heat-treated samples, *ACT2* and *EF1α* were identified as the most stable reference genes by all three of the algorithms. The agreement among algorithms in classifying the suitability of reference genes has been also documented in peach [33], cucumber [11] and litchi [53]. Indeed, as has also been noted by many authors [22], [34], the use of more than one algorithm leads to highly correlated results, especially regarding the most and the least stable reference genes, and represents a good strategy for the selection of reference genes for qPCR normalization. Nevertheless, differences in the ranking order of the most stably expressed genes for the NaCl-treated samples among the three software packages have also been demonstrated by other studies [22], [54], [55], although the two least variable reference genes identified by each algorithm were very similar. This variation was unsurprising, as different algorithms and analytical procedures are used in the three software packages and do not seem to affect the overall validation quality. Comparable results have also been encountered and discussed in numerous previous studies [24], [26], [56]. Taken together, the results obtained from the three software packages identified *EF1α* and *β-TUB* as the overall optimum pair of reference genes across all the samples, abiotic stress and hormone treatments and different tissue types. These results also suggested that the stability of candidate reference genes must be evaluated under different experimental conditions prior to gene expression normalization.

Our data demonstrated that *EF1α* and *β-TUB*, followed by *EIF4* and *CYP*, were the top four reference genes across all the samples, as well as the abiotic stress samples, in maize. In the current study, *EF1α* was ranked as one of the top two reference genes, which is consistent with the results in potato [31], rice under biotic and abiotic stress [19], soybean [18], longan tree [57], *Lolium temulentum* [58], and perennial ryegrass [59]; however, the gene performed poorly in studies of *Nicotiana benthaminana* [60], *Platycladus orientalis* [5], soybean [19], wheat [22], and tomato [27], suggesting that the expression levels of reference genes are variable among species. *β-TUB*, another of the most stable reference genes in our study, was also the best performer among different tissues and PEG-treated samples of *Platycladus orientalis* [5], across various developmental stages of soybean [19] and in different tissues of poplar [25], but it was the worst performer in *Brachiaria brizantha* [61], rice [19], and citrus [26]. *EIF4* showed remarkably consistent expression in papaya [62], *Brachiaria brizantha* [61], and grape [34]; this was also the case in the samples treated by cold and NaCl analyzed by BestKeeper in the current study. However, there is also evidence that the expression profile of this gene is not as consistent as those of other tested reference genes [19], [59]. In addition, our result is similar to Nicot et al., who found that *CYP* was the most stable gene in potato under salt stress [31] but the least stable in citrus subjected to biotic stress [26].

The expression levels of the reference genes *ACT2*, *UBQ7*, and *GAPDH* were rather variable in this study. For example, *ACT2* expressed most stably in the heat- and NaCl-treated sample sets but was only moderately stable in the total sample set and the abiotic stressed sample set; the gene was identified as the least stable in the different tissues by geNorm analysis. *UBQ7* performed best in the different tissues according to geNorm but worst in the NaCl-treated samples according to BestKeeper. *GAPDH* was identified as the best gene in the NaCl-treated samples by geNorm, in the PEG-treated samples and across different tissues by NormFinder and in the PEG-treated samples by BestKeeper. However, the gene was identified as a poor performer in the cold-treated samples by geNorm, in the cold- and heat-treated samples by NormFinder, and in the heat- and PEG-treated samples in BestKeeper. Similar results have also been found in many previous studies [2], [4], [5], [11].

Conversely, *GRP*, *GLU1*, and *UBQ9* were ranked at the bottom positions in this study. *UBQ*9 has previously been reported as stably expressed in NaCl- and ABA-treated samples of *Platycladus orientalis* [5], peach [33], rice[19], and *Brassica juncea* [4]. However, in this study, all three algorithms ranked *UBQ9* as among the most unstable reference genes. Similarly, the novel reference gene *GLU1* was one of the least stable reference genes across all the experimental sets. These results indicate that *UBQ* and *GLU1* are unsuitable for most sample treatments in maize. Additionally, *GRP* was the lowest-ranked gene across most of the experimental condition sets, although it outperformed other reference genes in the samples from different tissues in maize. Therefore, to confirm the transcript stability of the commonly used reference genes and to identify novel or superior reference genes, it is necessary to collect as much data as possible about gene expression in different organisms, organs, and experimental conditions.

In this study, we also used the potential stable reference genes *EF1a* and *β-TUB* and their combination (*EF1a*+*β –TUB*) to normalize the expression of *WRKY50* in maize. The results showed that *WRKY50* expression was induced by heat, NaCl, cold, and SA when compared with the control group, suggesting a more general role for *WRKY50* in maize. The *WRKY50* expression profile in our study was consistent with that reported in *Arabidopsis* [2]. Meanwhile, the tissue-specific expression patterns of *WRKY50* were similar to those of a previous report in maize across different development stages [47]. Our results further confirmed that the most stable reference genes (*EF1α* and *β-TUB*) identified in our study could be used for the accurate normalization of gene expression in maize under the experimental conditions tested here.

It is worth noting that although Manoli *et al.* [43] evaluated the candidate reference genes in maize by similar approaches, they focused on exploring novel reference genes but not evaluating traditional ones which were assessed in this study. Furthermore, the candidate reference genes were evaluated by Manoli *et al.* [43] under experimental conditions such as +N/−N nutrient, day/night cycle, darkness, and high temperature. In contrast, our evaluation on these reference genes was carried out under different treatments of abiotic stresses (salt, heat shock, cold, and PEG), hormones, and tissue types. In addition, the selected candidate reference genes were confirmed afterwards by normalization the expression of target gene *WRKY50* under various experimental conditions in this study, which is a good example for further application of these reference genes.

In summary, this article describes a systematic attempt to validate a set of commonly used candidate reference genes for the normalization of gene expression using RT-qPCR in maize samples subjected to five abiotic stresses and five hormone treatments and across different tissue types. Evaluations using geNorm, NormFinder, and BestKeeper identified the four most suitable reference genes in maize as *EF1a*, *β-TUB*, *EfcgIF4*, and *CYP* and the three least suitable reference genes as *GRP*, *GLU1*, and *UBQ9*; these genes may be unsuitable for future maize studies. Further validation using each of the most stable reference genes, *EF1a* and *β-TUB*, and their combination (*EF1a*+*β-TUB*) confirmed that *EF1a* and *β-TUB* were the appropriate reference genes for the normalization of RT-qPCR data, and the combination of more than one reference gene was recommended.

## Supporting Information

Figure S1 **PCR production of the reference gene primers.**
(DOCX)

Figure S2 **Melt curves of the ten candidate reference genes.**
(DOC)

Figure S3 **Standard curves of ten candidate reference genes.**
(DOC)

Figure S4 **Melt curve and standard curve of** ***WRKY50*** **.**
(DOC)

Table S1 **Candidates reference genes ranked according to their expression stability value (M) estimated using geNorm algorithm.**
(DOC)

Table S2 **Primer sequences, product sizes and amplicon characteristics of** ***WRKY50*** **.**
(DOCX)

Text S1 **PCR product of ten candidate reference genes.**
(DOCX)
