# Supplementary material for: Validation of Potential Reference Genes for qPCR in Maize across Abiotic Stresses, Hormone Treatments, and Tissue Types
Source: PLoS One. 2014 May 8;9(5):e95445. doi: 10.1371/journal.pone.0095445 (PMC4014480; doi:10.1371/journal.pone.0095445)
Supplement: Figure S1 — PCR production of the reference gene primers. (DOCX) [file pone.0095445.s001.docx]

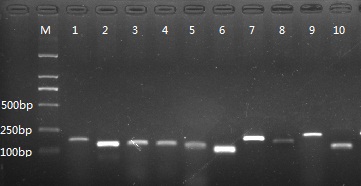


**Figure S1. PCR production of the reference gene primers**

M: DNA Maker; 1: *GAPDH*; 2: *EF1α*; 3: *β-TUB*; 4: *ACT2*; 5: *UBQ9*;

6: *CYP*; 7: *EIF4A*; 8: *UBQ7*; 9: *GLU1*; 10: *GRP*
