# Supplementary material for: Validation of Potential Reference Genes for qPCR in Maize across Abiotic Stresses, Hormone Treatments, and Tissue Types
Source: PLoS One. 2014 May 8;9(5):e95445. doi: 10.1371/journal.pone.0095445 (PMC4014480; doi:10.1371/journal.pone.0095445)
Supplement: Figure S2 — Melt curves of the ten candidate reference genes. (DOC) [file pone.0095445.s002.doc]

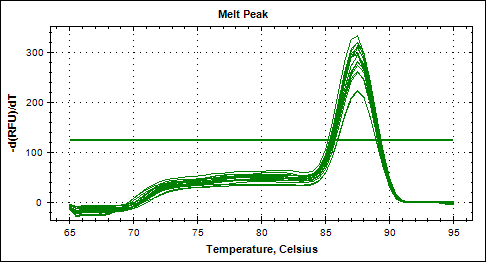

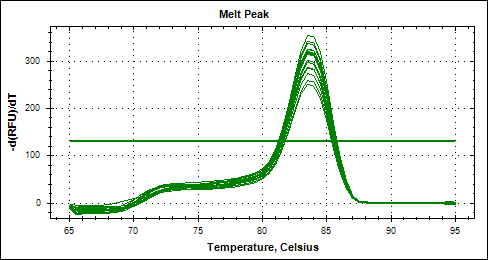


A

B


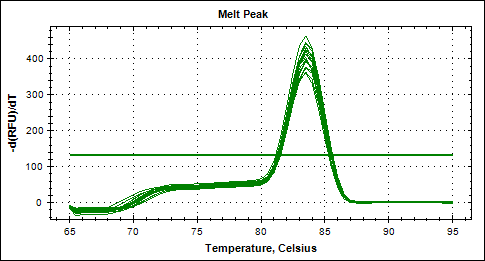

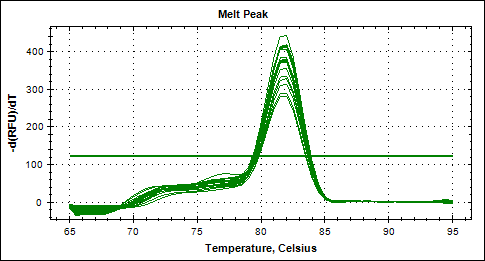


C

D


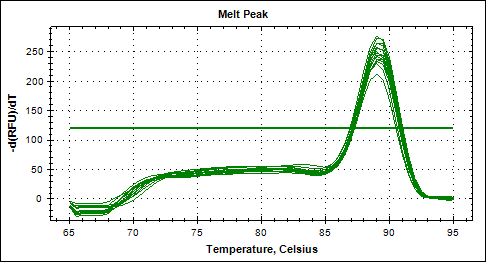

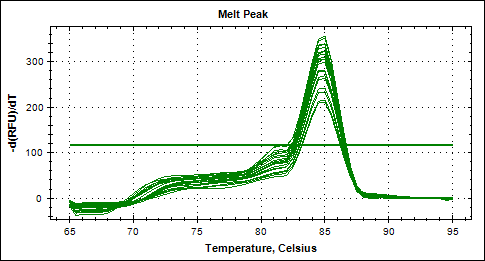


E

F


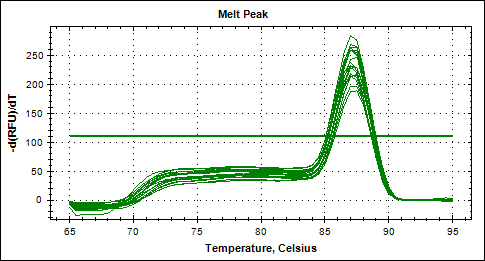

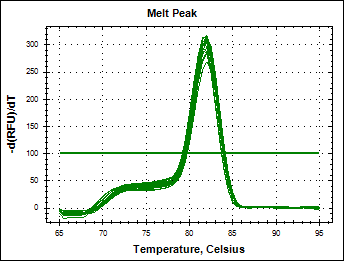


G

H


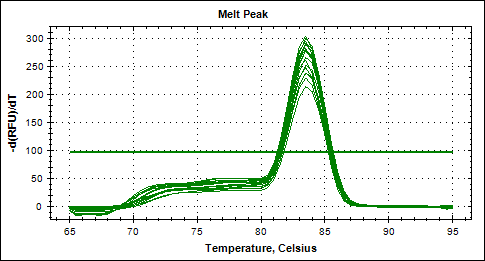

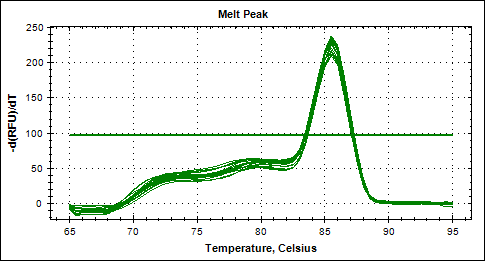


I

J

**Figure S2. Melt curves of the ten candidate reference genes**

A:*GAPDH* B:*EF1a* C:*β-TUB* D:*ACT2* E:*UBQ9* F:*GLU1* G:*GRP* H:*CYP* I:*EIF4A* J:*UBQ7*
