# Supplementary figures and images for: Validation of Potential Reference Genes for qPCR in Maize across Abiotic Stresses, Hormone Treatments, and Tissue Types
Source: PLoS One. 2014 May 8;9(5):e95445. doi: 10.1371/journal.pone.0095445 (PMC4014480; doi:10.1371/journal.pone.0095445)

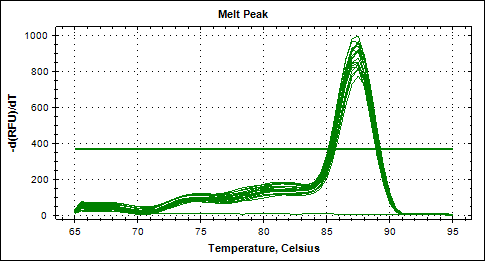

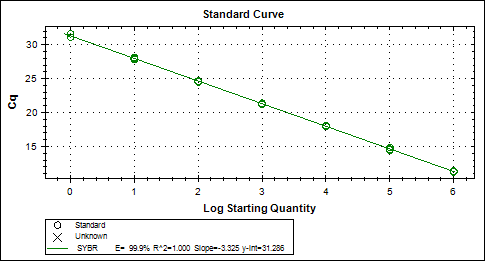


A

B

**Figure S4 Melt curve and standard curve of *WRKY50***

1. melt curve; (B) standard curve

Supplement: Figure S4 — Melt curve and standard curve of WRKY50 . (DOC) [file pone.0095445.s004.doc]
