# Supplementary material for: Validation of Potential Reference Genes for qPCR in Maize across Abiotic Stresses, Hormone Treatments, and Tissue Types
Source: PLoS One. 2014 May 8;9(5):e95445. doi: 10.1371/journal.pone.0095445 (PMC4014480; doi:10.1371/journal.pone.0095445)
Supplement: Table S1 — Candidates reference genes ranked according to their expression stability value (M) estimated using geNorm algorithm. (DOC) [file pone.0095445.s005.doc]

Table S1 Candidates reference genes ranked according to their expression stability value (M) estimated using geNorm algorithm.

| Rank | Total | | Cold | | Heat | | PEG | | NaCl | | Tissue | | Hormone | | Abiotic stresses | |
| --- | --- | --- | --- | --- | --- | --- | --- | --- | --- | --- | --- | --- | --- | --- | --- | --- |
|  | *EF1α* |  | *CYP* |  | *EF1α* |  | *EF1α* |  | *GAPDH* |  | *GRP* |  | *EF1α* |  | *EF1α* |  |
| 1 | *β-TUB* | 0.485 | *EIF4A* | 0.114 | *ACT2* | 0.214 | *β-TUB* | 0.268 | *ACT2* | 0.431 | *UBQ7* | 0.05 | *β-TUB* | 0.125 | *β-TUB* | 0.549 |
| 2 | *UBQ9* | 0.646 | *EF1a* | 0.359 | *CYP* | 0.427 | *GAPDH* | 0.304 | *EF1a* | 0.7 | *CYP* | 0.242 | *EIF4A* | 0.347 | *GLU1* | 0.646 |
| 3 | *EIF4A* | 0.757 | *GLU1* | 0.42 | *EIF4A* | 0.555 | *UBQ9* | 0.331 | *β-TUB* | 0.76 | *GAPDH* | 0.36 | *CYP* | 0.464 | *UBQ9* | 0.708 |
| 4 | *CYP* | 0.805 | *UBQ7* | 0.448 | *β-TUB* | 0.644 | *ACT2* | 0.38 | *UBQ7* | 0.83 | *EIF4A* | 0.578 | *UBQ7* | 0.507 | *CYP* | 0.791 |
| 5 | *GAPDH* | 0.886 | *ACT2* | 0.57 | *GLU1* | 0.707 | *UBQ7* | 0.504 | *CYP* | 0.901 | *EF1a* | 0.676 | *GAPDH* | 0.602 | *EIF4A* | 0.835 |
| 6 | *ACT2* | 0.937 | *β-TUB* | 0.621 | *UBQ9* | 0.787 | *CYP* | 0.588 | *EIF4A* | 0.968 | *β-TUB* | 0.716 | *GRP* | 0.636 | *GAPDH* | 0.894 |
| 7 | *UBQ7* | 0.986 | *UBQ9* | 0.658 | *GAPDH* | 0.858 | *GRP* | 0.659 | *GLU1* | 1.027 | *GLU1* | 0.785 | *ACT2* | 0.662 | *ACT2* | 0.934 |
| 8 | *GLU1* | 1.039 | *GAPDH* | 0.703 | *UBQ7* | 0.942 | *GLU1* | 0.726 | *GRP* | 1.071 | *UBQ9* | 0.889 | *UBQ9* | 0.716 | *UBQ7* | 0.997 |
| 9 | *GRP* | 1.281 | *GRP* | 1.11 | *GRP* | 1.544 | *EIF4A* | 0.797 | *UBQ9* | 1.136 | *ACT2* | 0.983 | *GLU1* | 0.873 | *GRP* | 1.297 |

Supplementary table 2 Pairwise variation (V_n_/V_n+1)_ of reference genes using geNorm algorithm

|  | V_2/3_ | V_3/4_ | V_4/5_ | V_5/6_ | V_6/7_ | V_7/8_ | V_8/9_ | V_9/10_ |
| --- | --- | --- | --- | --- | --- | --- | --- | --- |
| Total | 0.23 | 0.195 | 0.151 | 0.153 | 0.128 | 0.121 | 0.116 | 0.215 |
| Cold | 0.159 | 0.107 | 0.088 | 0.128 | 0.095 | 0.083 | 0.087 | 0.272 |
| Heat | 0.174 | 0.16 | 0.142 | 0.124 | 0.13 | 0.119 | 0.124 | 0.392 |
| PEG | 0.098 | 0.082 | 0.084 | 0.119 | 0.104 | 0.097 | 0.099 | 0.096 |
| NaCl | 0.271 | 0.185 | 0.173 | 0.148 | 0.147 | 0.136 | 0.112 | 0.127 |
| Tissue | 0.112 | 0.117 | 0.179 | 0.144 | 0.106 | 0.112 | 0.135 | 0.125 |
| Hormone | 0.152 | 0.136 | 0.099 | 0.122 | 0.09 | 0.076 | 0.092 | 0.143 |
| Abiotic stresses | 0.213 | 0.17 | 0.165 | 0.129 | 0.128 | 0.11 | 0.119 | 0.242 |
