# Supplementary material for: Validation of Potential Reference Genes for qPCR in Maize across Abiotic Stresses, Hormone Treatments, and Tissue Types
Source: PLoS One. 2014 May 8;9(5):e95445. doi: 10.1371/journal.pone.0095445 (PMC4014480; doi:10.1371/journal.pone.0095445)
Supplement: Table S2 — Primer sequences, product sizes and amplicon characteristics of WRKY50 . (DOCX) [file pone.0095445.s006.docx]

| Gene symbol | Accession  number | Primer sequence (5’–3’) | Tm(℃) | Size (bp) | PCR efficiency | R^2^ |
| --- | --- | --- | --- | --- | --- | --- |
| *WRKY50* | GRMZM2G071907 | F: CGTGAAGAGGACCATCAGAGTG  R: CTTGTAGTAGCCCCGTGGGTAAG | 62.2  63.1 | 130 | 99.9% | 1.000 |

**Table S2 primer sequences, product size and amplicon characteristics of *WRKY50***
