# Supplementary material for: Validation of Potential Reference Genes for qPCR in Maize across Abiotic Stresses, Hormone Treatments, and Tissue Types
Source: PLoS One. 2014 May 8;9(5):e95445. doi: 10.1371/journal.pone.0095445 (PMC4014480; doi:10.1371/journal.pone.0095445)
Supplement: Text S1 — PCR product of ten candidate reference genes. (DOCX) [file pone.0095445.s007.docx]

**Text 1 PCR product of ten candidate reference genes**

*ACT2*

>*ctgaggttctattccagccatccttcattggcatggaatctgctggcatccatgaagccacgtacaactccatcatgaagTgcgacgtcgatatcagaaaggatctgtacggtaatgttgtcctcagtggtgg*

*GAPDH*

>*ccatcactgccacacagaaaactgttgacggaccctcagccaaggactggagaggtggcagggctgccagctttaacatcattcctagcagcaccggtgctgccaaggctgttggtaaagttcttcctgatctgaatggcaagctcactggtatgtccttccgtgttcct*

*EF1á*

*>tgggcctactggtcttactactgaggttaagtccgttgagatgcaccatgaggctctccaggaggcccttcccggtgacaatgttggcttcaacgtgaagaatgttgctgtgaaggatctgaagcgtgggtatgt*

*â-TUB*

*>ctacctcacggcatctgctatgttccgtgggaagatgagcaccaaggaagtggatgaacagatgctgaacgtgcagaacaagaactcttcctacttcgtggagtggatccccaacaacgtgaagtcgagtgtgtgtgac*

*UBQ9*

>*tgcagttctacaaggtggacgacgccaccggcaaggtgacccgcctccgcaaggagtgccccaacaccgagtgcggtgcgggtgtcttcatggccaaccacttcgaccgccactactgc*

*CYP*

>*ctgagtggtggtcttagtttatcatgtatcgctcgcagtttaatttagcggtttaggtgtggatctgtgaaccccatggcgcctctgcttgattcgtgtt*

*EIF4A*

*>cgtccagaggttctacaacgtgaccgttgaggagctgccggccaatgtcgccgaccttctctagatagtatggctgctaaggtttctggataaaggagtttgtttttgttaaaatacgccaacttttctctgctttggatgagtggtaaggtccggggtctgttcgtattgtggcgaaggatg*

*UBQ7*

*>cagactacaacatccagaaggagagcactctccacctggtgctccgcctccgcggtggtcagtaagccatggttcatttaagctgctgctgtacctgggtatctgcgtcgtctggtgccctctggtgtacctctatatggatgtcgtcgtctaata*

*GLU1*

*>ATGAAGGAGTCTGCCAAGTGGTTGAAAGAGTTCAACACCGCGAAAAAGCCCAGCAAGAAGATTCTTACGCCAGCTTAAAAATCGGGGGCCTCATGATGTGGGTGCAGCCCATAAAAACTGTGTGGTTTGGAACCGAAGATTTTCTCTTTTTTTTTCTGCCACGAGAGGTTCTCTGGAGGCATACTCTCCAGCACCG*

*GRP*

*>AACGAGTCGCTGGAGAATGCCTTCGCCTCCTACGGCGAGATCCTCGACTCCAAGGTCATCACCGACCGGGAGACGGGGAG GTCCCGCGGCTTCGGCTTCGTTACCTTCTCCTCCGA*
